# Knowledge of and Compliance With Guidelines in the Management of Non-Muscle-Invasive Bladder Cancer: A Survey of Chinese Urologists

**DOI:** 10.3389/fonc.2021.735704

**Published:** 2021-10-27

**Authors:** Dan-Qi Wang, Qiao Huang, Xing Huang, Ying-Hui Jin, Yun-Yun Wang, Yue-Xian Shi, Si-Yu Yan, Lu Yang, Bing-Hui Li, Tong-Zu Liu, Xian-Tao Zeng

**Affiliations:** ^1^ Country Center for Evidence-Based and Translational Medicine, Zhongnan Hospital of Wuhan University, Wuhan, China; ^2^ Department of Urology, Institute of Urology, Zhongnan Hospital of Wuhan University, Wuhan, China; ^3^ School of Nursing, Peking University, Beijing, China; ^4^ Department of Urology, Institute of Urology, West China Hospital of Sichuan University, Chengdu, China

**Keywords:** guideline, guideline adherence, urinary bladder neoplasms, surveys and questionnaires, professional practice

## Abstract

**Background:**

Non-muscle-invasive bladder cancer (NMIBC) still poses a heavy load for resulting in many new cases which contribute significantly to medical costs. Although many NMIBC guidelines have been developed, their implementation remains deficient.

**Objective:**

This study was conducted in order to analyze the knowledge of and compliance with the guidelines for NMIBC of Chinese urologists and to identify associated factors.

**Methods:**

We conducted an online survey between August 2019 and January 2021. Respondents who were more than 65 years old or did not give informed consent were excluded. Linear/logistic regressions were performed to identify factors associated with the knowledge of and compliance with the guidelines of urologists, respectively. McNemar’s tests were used to explore the divergence between knowledge and compliance.

**Results:**

A total of 814 responses were received, and 98.77% of urologists acknowledged the positive effects of high-quality guidelines. The average knowledge score was 6.10 ± 1.28 (out of a full score of 9), and it was positively associated with educational level and the number of guidelines consulted. Only 1.61% and 39.36% of the respondents realized that the guidelines did not recommend further chemotherapy or BCG infusion for low-risk patients. There were 38.87% and 51.84% respondents “often” or more frequently utilizing BCG therapy for intermediate- and high-risk NMIBC patients, respectively. Divergence between knowledge and compliance in performing a second TURBT after incomplete initial resection reached statistical significance (*p* < 0.001).

**Conclusions:**

Although the vast majority of urologists acknowledged the positive effects of guidelines, knowledge of and compliance with some recommendations of NMIBC guidelines are still inadequate. Factors associated with guidelines, individual professionals, patients, organizations, and the environment jointly contributed to the non-compliance.

## Introduction

Ranking as the 10th most common cancer worldwide, bladder cancer caused an estimated 549,000 new cases and 200,000 deaths worldwide in 2018 (1). For therapeutic purposes, papillary tumors confined to the mucosa (Ta) or submucosa (T1) and carcinoma *in situ* (CIS, Tis) are classified as non-muscle-invasive bladder cancer (NMIBC) (2), which accounts for approximately 75% of bladder cancer cases (3). NMIBC still poses a heavy load due to its high 5-year recurrence (up to 78%) and progression (up to 45%) risk (4), hence incurring high medical costs.

For the universal coverage of high-quality medical services, institutions and academies worldwide have invested substantial resources in developing treatment guidelines. The European Association of Urology (EAU), National Comprehensive Cancer Network (NCCN), and American Urological Association (AUA) have published guidelines relating to NMIBC, and all of them updated their guidelines last year. However, although the positive effects of guidelines have been demonstrated (5, 6), many studies have reported that guideline-prescribed care was not universally introduced into clinical management of NMIBC (5, 7, 8), especially the use of intravesical adjuvant therapy (8–10).

The knowledge, attitude, and behavior of urologists have a great impact on the process from guideline to practice (11). However, currently, there are insufficient studies on variations in quality of bladder cancer care, and limited attention has been paid to barriers that urologists meet with in clinical practice. In this study, we collected responses of urologists with the following aims: i) to identify the knowledge and compliance rate with NMIBC guidelines; ii) to reveal the variation between knowledge and compliance, guideline, and clinical practice; and iii) to find reasons and explanations for non-compliance. To remove the impact of preference of the patient, the compliance with the guidelines of a urologist was defined as the extent to which his clinical suggestion coincided with guideline recommendations (12). It was noted that guidelines did not have any legislative influence or override the responsibility to make decisions.

## Methods

### Study Design

Applying convenience and snowball sampling, we conducted an online survey on the knowledge of and compliance with NMIBC guidelines of Chinese urologists from October 2019 to January 2021. The questionnaire link was sent *via* a QR code posted on several national urological specialist forums in China. Meanwhile, we requested respondents to help us disseminate the questionnaire. Information for informed consent included the statement that individual responses would remain anonymous. Respondents who were more than 65 years old or did not give informed consent were excluded. No incentive was offered for participation.

### Development of the Questionnaire

An exhaustive online literature review of NMIBC guidelines worldwide was conducted. We had appraised the quality of NMIBC guidelines within the past 5 years and compared the similarities and differences of therapeutic recommendations between guidelines previously (13). Based on this, six core interventions pertaining to the management of NMIBC were formulated, which addressed the initial transurethral resection of bladder tumor (TURBT), second resection, immediate postoperative intravesical instillation of chemotherapy, additional adjuvant intravesical chemotherapy instillations, intravesical bacillus Calmette–Guerin (BCG) immunotherapy, and radical cystectomy. After several rounds of group meetings, the draft was sent to three clinical urological experts and two methodologists for consultation to verify the face validity. Based on the valuable opinions of experts, we deleted the item of “device-assisted chemotherapy” and added the interpretation for “BCG failure.” Through these efforts, we enhanced the acceptability and clarity of the questionnaire and also improved its expression to promote understanding.

Finally, the questionnaire consisted of the following six sections: i) demographic characteristics of urologists; ii) attitude toward guidelines in general, where the information of preferred guideline developers were gathered; iii) clinical utilization of chemotherapy and BCG; iv) knowledge of guidelines, in which the knowledge score of recommendations in NMIBC guidelines was assessed by nine items, while each item scored 1 point. Regarding the indications for surgery, given that all of them were extracted from the guidelines, 1 score was divided equally among each option (for example, if there were five indications for a second TURBT, then 0.2 point was given for each indication selected); v) compliance with guidelines, which focused on the strategy of chemotherapy and BCG for patients of each risk classification (low risk, intermediate risk, and high risk); and vi) barriers in clinical practice, which involved the barriers that respondents met in the clinical practice of a second TURBT, BCG, and radical cystectomy (**Appendix 1**). Urologists were asked to use yes/no/unsure or check choices of medical interventions to indicate whether they thought a medical intervention was recommended by guidelines or whether they would comply with it. Questions about barriers to guideline implementation were multiple choice. When asking about frequency, Likert-scale questions were adopted on a 5-point scale as never, seldom, sometimes, often, and always. Text forms were offered where extra information was needed.

### Statistical Analysis

Descriptive statistics were used to summarize the data, where categorical variables were expressed as counts and percentages, and continuous variables were expressed as mean and standard deviation. McNemar’s tests were used to compare the knowledge and compliance in each item. To identify characteristic factors associated with knowledge, we performed univariable and multivariable linear regressions. While univariable and multivariable logistic regressions were conducted for factors associated with compliance, Firth’s logistic regressions were applied for unbalanced event with potential complete or quasi-complete separation issue. Statistical analyses were performed using SAS software, version 9.4 TS1M6 (SAS Institute Inc., Cary, NC), and a two-sided *p*-value of ≤0.05 was considered statistically significant.

## Results

### Characteristics of the Respondents

A total of 814 responses were received, 2 of which were excluded for overage and informed disconsent, respectively. The vast majority of respondents were working in tertiary hospitals (78.45%), had been in practice for more than 15 years (63.18%), and had a master’s degree or above (56.28%) (**Table 1**). Although only 10.71% respondents reported having had prior experience in guideline development, most respondents (98.77%) agreed that high-quality guidelines were efficacious in improving healthcare quality, standardizing the clinical procedures and quality assurance. Respondents preferred guidelines developed by the Chinese Medical Association (CMA) (72.78%), European Association of Urology (EAU) (60.96%), and American Urological Association (AUA) (48.77%). Respondents usually referred to an average of 2.78 ( ± 1.46) NMIBC guidelines.

**Table 1:** Characteristics of the respondents.

	*n*	%
Gender		
Male	796	98.03%
Female	16	1.97%
Age		
<40	285	35.10%
40–59	515	63.42%
≥60	12	1.48%
Years of clinical practice		
<15	299	36.82%
15–29	404	49.75%
≥30	109	13.42%
Level of healthcare institute		
3	637	78.45%
2	174	21.43%
1	3	0.37%
Education background		
Doctor	195	24.01%
Master	262	32.27%
Bachelor	345	42.49%
College	10	1.23%
Professional title		
Senior title	233	28.69%
Vice-senior title	299	36.82%
Middle title	119	14.66%
Primary title	61	7.51%
Preferred guideline developer		
Chinese Medical Association (CMA)	591	72.78%
European Association of Urology (EAU)	495	60.96%
American Urological Association (AUA)	396	48.77%
National Comprehensive Cancer Network (NCCN)	298	36.70%
Canadian Urological Association (CUA)	267	32.88%
Chinese Medical Doctor Association (CMDA)	236	29.06%
National Institute for Health and Care Excellence (NICE)	86	10.59%
Chinese Research Hospital Association (CRHA)	51	6.28%
Have participated in guideline development	87	10.71%
Recognized the positive function of guidelines	802	98.77%

### Clinical Utilization of Chemotherapy and BCG

There were 79.25% of the respondents who reported that low-risk NMIBC patients agreed to have chemotherapy for immediate course together with maintenance course. Respondents who reported that they “often” or more frequently utilized BCG therapy for intermediate- and high-risk NMIBC patients were only 38.87% and 51.84%, respectively.

For patients from each risk classification, at least 49.32% respondents scheduled induction course of intravesical chemotherapy instillations of 4 to 8 weeks, and 47.62% or more respondents scheduled a maintenance course of 6 to 12 months (**Appendix 2**). As for BCG, many respondents (from 42.80% to 48.14%) scheduled induction course of BCG instillations of 6 to 8 weeks, while a maintenance course of 4 weeks or more was chosen by at least 61.98% respondents. The median length of maintenance installations was 1 year. Moreover, a majority of respondents (from 76.39% to 90.21%) approved of a standard dose of BCG.

### Knowledge of Guideline Recommendations

The average knowledge score of respondents was 6.10 ± 1.28 (out of a full score of 9). “The range of initial TURBT” (97.40%) and “whether to perform an immediate postoperative instillation” (96.77%) got the highest correct rate (**Table 2**). However, for low-risk patients, only 3.97% respondents realized that further chemotherapy was not recommended, and 43.08% respondents recognized that BCG immunotherapy was also not recommended.

**Table 2:** Knowledge of and compliance with guideline recommendations.

No.	Recommendations	*N* (%)		*p*-value
		Knowledge	Compliance	
1	The presence of detrusor muscle in the specimen is necessary for the NMIBC patients having had initial TURBT	786 (97.40)	/	/
2	Perform a single postoperative instillation of intravesical chemotherapy after TURBT	780 (96.77)	/	/
	Further chemotherapy instillations			
3	Not recommended for low-risk NMIBC patients	32 (3.97)	13 (1.61)	<0.001
4	Provide induction and maintenance chemotherapy instillations for intermediate-risk NMIBC patients	735 (91.99)	743 (92.99)	0.243
5	Provide induction and maintenance chemotherapy instillations for high-risk NMIBC patients	/	704 (89.00)	/
	BCG immunotherapy			
6	Not recommended for low-risk NMIBC patients	336 (43.08)	307 (39.36)	0.181
7	Provide induction and maintenance chemotherapy instillations for intermediate-risk NMIBC patients	513 (66.97)	518 (67.62)	0.685
8	Provide induction and maintenance chemotherapy instillations for high-risk NMIBC patients	608 (79.89)	614 (80.68)	0.451
9	Indications for a second TURBT			
	After incomplete initial TURBT	640 (79.60)	542 (67.41)	<0.001
	There is no muscular layer in the first resected specimen (except for TaLG/G1 tumor and carcinoma *in situ*)	598 (74.38)	595 (74.00)	0.863
	In T1 tumors	347 (43.16)	357 (44.40)	0.474
	In G3/high-grade tumors (except for CIS)	456 (56.72)	441 (54.85)	0.228
	Pathology analysis results of initial TURBT failed to determine stage or risk grading	485 (60.32)	443 (55.10)	0.001
10	Indications for radical cystectomy			
	High-grade T1 with histological variation (micropapillary, sarcoma, small cell type)	466 (58.54)	398 (50.00)	<0.001
	High-grade T1 with lymphatic vessel infiltration, multiple and/or large high-grade T1, high-grade T1 with bladder/prostate CIS	654 (82.16)	634 (79.65)	0.111
	Pathology analysis results of a second TURBT is still high-grade T1	491 (61.68)	545 (68.47)	<0.001
	High-grade NMIBC with early recurrence within 3 months	532 (66.83)	542 (68.09)	0.440
	NMIBC involving the bladder diverticulum	331 (41.58)	384 (48.24)	<0.001
	High-risk NMIBC patients with BCG failure	590 (74.12)	526 (66.08)	<0.001

### Compliance With Guideline Recommendations

There were 98.39% of the respondents who tended to utilize further chemotherapy instillations for patients with low-risk NMIBC (**Table 2**). Notably, the difference between the knowledge and compliance was statistically significant in items of further chemotherapy for low-risk patients (*p*_low_ < 0.001). For intermediate-risk patients, although BCG was widely recommended in the guidelines, the compliance rate (67.62%) was much lower than that for chemotherapy (92.99%). More respondents (89.00%) suggested further chemotherapy for high-risk patients even though it was not supported by many guidelines.

Divergence between knowledge rate and compliance rate also existed in some items of surgery indications. In a second TURBT, the compliance rate was relatively lower than knowledge rate for the indication of “after incomplete initial TURBT” (79.60% vs. 67.41%, *p* < 0.001), so did “pathology analysis results of initial TURBT failed to determine stage or risk grading” (60.32% vs. 55.10%, *p* < 0.0011).

### Characteristic Factors Associated With Knowledge and Compliance

We used multiple linear regressions to assess characteristic factors associated with knowledge score of the respondents (**Table 3**). Knowledge scores were positively and independently associated with the number of guidelines that respondents usually referred to, and the adjusted coefficient was 0.17 (95% CI, 0.10 to 0.23), *p* < 0.001. Compared with bachelors, masters and PhDs were more likely to gain higher knowledge scores (*B* = 0.67, *p* < 0.001; *B* = 0.42, *p* < 0.001). Besides, respondents from the Middle may gain lower scores than those from the East and the Northeast (we combined the data of respondents from these two regions for regression analysis) (*B* = −0.28, *p* = 0.022).

**Table 3:** Multivariable linear regressions of knowledge of guidelines.

Variables	Level	Multivariable	
		Coefficient (95% CI)	*p*-value
Hospital level	Tertiary	0.20 (−0.06, 0.46)	0.128
	Secondary and below	Reference	
Years of practice	11~20	0.12 (−0.16, 0.39)	0.401
	21~30	−0.05 (−0.40, 0.30)	0.786
	≥31	−0.14 (−0.60, 0.32)	0.551
	≤10	Reference	
Education background	PhD	0.67 (0.39, 0.95)	<0.001
	Master	0.67 (0.39, 0.95)	<0.001
	Bachelor or college	Reference	
Professional title	Senior	0.30 (−0.05, 0.66)	0.094
	Vice-senior	0.12 (−0.15, 0.38)	0.391
	Middle and below	Reference	
Having participated in guideline development	Yes	−0.00 (−0.30, 0.29)	0.973
	No	Reference	
Region	The Middle	−0.28 (−0.51, −0.04)	0.022
	The West	−0.10 (−0.33, 0.14)	0.431
	The East and the Northeast	Reference	
Number of guidelines consulted		0.23 (0.07, 0.39)	0.005

CI, confidence interval.

Univariable and multivariable logistic regressions were performed to identify factors associated with the compliance of the respondent with guideline recommendation (**Tables 4**, **5**). Knowledge of the recommendation was demonstrated to affect the compliance positively (*p* < 0.001). In some items, respondents who consulted more guidelines were more likely to comply with the guidelines, such as further chemotherapy and BCG therapy for patients with intermediate-risk NMIBC (OR = 1.32, *p* = 0.047; OR = 1.22, *p* = 0.015). However, the result of BCG therapy for low-risk patients was reversed (OR = 0.87, *p* = 0.034). Regarding further chemotherapy for intermediate-risk patients (OR = 0.24, *p* = 0.033) and BCG for high-risk patients (OR = 0.36, *p* = 0.016), respondents from the Middle were more likely to gain less score than those from the East and the Northeast.

**Table 4:** Multivariable logistic regression of compliance with recommendation about chemotherapy.

Independent variables	Level	Dependent variables					
		Low-risk patients		Intermediate-risk patients		High-risk patients	
		OR (95% CI)	*p*-value	OR (95% CI)	*p*-value	OR (95% CI)	*p*-value
Hospital level	Tertiary	1.93 (0.08, 45.97)	0.684	0.38 (0.13, 1.11)	0.077	1.80 (0.63, 5.15)	0.272
	Secondary and below	Reference					
Years of practice	11~20	0.24 (0.03, 2.21)	0.207	1.77 (0.54, 5.86)	0.348	0.46 (0.16, 1.33)	0.15
	21~30	0.22 (0.02, 3.20)	0.271	0.87 (0.18, 4.23)	0.86	1.24 (0.29, 5.38)	0.773
	≥31	0.20 (0.01, 4.52)	0.31	0.51 (0.07, 3.58)	0.496	1.07 (0.18, 6.51)	0.942
	≤10	Reference					
Education background	PhD	4.42 (0.37, 52.56)	0.239	5.76 (1.30, 25.49)	0.021	2.48 (0.67, 9.16)	0.174
	Master	6.11 (0.65, 57.62)	0.114	1.23 (0.47, 3.21)	0.674	0.94 (0.36, 2.44)	0.901
	Bachelor or college	Reference					
Professional title	Senior	3.39 (0.25, 46.34)	0.361	3.25 (0.61, 17.31)	0.167	2.33 (0.53, 10.23)	0.263
	Vice-senior	0.71 (0.09, 5.50)	0.744	1.28 (0.41, 4.04)	0.671	2.91 (0.98, 8.62)	0.054
	Middle and below	Reference					
Having participated in guideline development	Yes	0.61 (0.11, 3.32)	0.566	0.15 (0.05, 0.47)	0.001	0.62 (0.16, 2.49)	0.503
	No	Reference					
Region	The Middle	1.12 (0.22, 5.60)	0.894	0.24 (0.07, 0.89)	0.033	1.14 (0.38, 3.47)	0.816
	The West	1.96 (0.48, 7.96)	0.347	0.39 (0.10, 1.46)	0.164	0.66 (0.22, 2.01)	0.464
	The East and the Northeast	Reference					
Number of guidelines consulted		1.12 (0.75, 1.68)	0.573	1.32 (1.00, 1.74)	0.047	0.79 (0.60, 1.05)	0.099
Knowledge of this recommendation		57.71 (16.77, 198.60)	<0.001	101.59 (44.85, 230.12)	<0.001	254.97 (104.95, 619.46)	<0.001

**Table 5:** Multivariable logistic regression of compliance with recommendation about BCG immunotherapy.

Independent variables	Level	Dependent variables					
		Low-risk patients		Intermediate-risk patients		High-risk patients	
		OR (95% CI)	*p*-value	OR (95% CI)	*p*-value	OR (95% CI)	*p*-value
Hospital level	Tertiary	0.88 (0.53, 1.47)	0.622	1.54 (0.81, 2.95)	0.19	2.85 (1.33, 6.12)	0.007
	Secondary and below	Reference					
Years of practice	11~20	1.51 (0.87, 2.60)	0.14	1.13 (0.56, 2.27)	0.734	0.77 (0.33, 1.82)	0.559
	21~30	1.08 (0.54, 2.16)	0.82	1.41 (0.58, 3.44)	0.449	0.49 (0.16, 1.50)	0.212
	≥31	1.06 (0.42, 2.67)	0.907	2.01 (0.62, 6.53)	0.243	0.50 (0.12, 2.05)	0.333
	≤10	Reference					
Education background	PhD	1.00 (0.57, 1.74)	0.991	0.92 (0.45, 1.88)	0.827	0.54 (0.21, 1.40)	0.204
	Master	1.30 (0.81, 2.08)	0.271	0.91 (0.50, 1.65)	0.76	0.53 (0.25, 1.10)	0.09
	Bachelor or college	Reference					
Professional title	Senior	0.96 (0.48, 1.92)	0.903	0.56 (0.23, 1.38)	0.206	1.03 (0.34, 3.08)	0.963
	Vice-senior	1.15 (0.69, 1.93)	0.593	0.64 (0.33, 1.26)	0.198	0.91 (0.41, 2.03)	0.813
	Middle and below	Reference					
Having participated in guideline development	Yes	0.67 (0.38, 1.20)	0.183	0.68 (0.33, 1.41)	0.3	1.57 (0.54, 4.50)	0.405
	No	Reference					
Region	The Middle	1.32 (0.82, 2.10)	0.251	0.66 (0.36, 1.20)	0.173	0.36 (0.16, 0.83)	0.016
	The West	1.35 (0.84, 2.17)	0.209	0.72 (0.38, 1.35)	0.301	0.70 (0.29, 1.65)	0.409
	The East and the Northeast	Reference					
Number of guidelines consulted		0.87 (0.77, 0.99)	0.034	1.22 (1.04, 1.43)	0.015	1.20 (0.99, 1.45)	0.068
Knowledge of this recommendation		10.47 (7.22, 15.17)	<0.001	38.01 (24.44, 59.12)	<0.001	77.17 (42.21, 141.08)	<0.001

### Barriers in Clinical Practice

There were 70.63% of the respondents who reported that “patients rejected the operation because of risk or side effects” prevented the implementation of a second TURBT in clinical practice, followed by “patients rejected the operation for economic reasons” (50.43%) and “urologist didn’t suggest it because of risk or complications” (37.55%) (**Appendix 3**). As data about the frequency of BCG utilization and radical cystectomy in clinical practice had been collected, we excluded respondents who “usually” or “always” implemented them in barrier analysis. For barriers that hindered the implementation of BCG, “drug was not accessible” was the dominant barrier (74.89%). Besides, there were 62.22% respondents who thought “patients rejected the operation for economic reasons” “often”, “usually”, or “always” hindered the implementation. With regard to radical cystectomy, 57.73% respondents indicated that “patients rejected it because of decrease in life quality” often or more frequently hindered the implementation.

**Figure 1:**
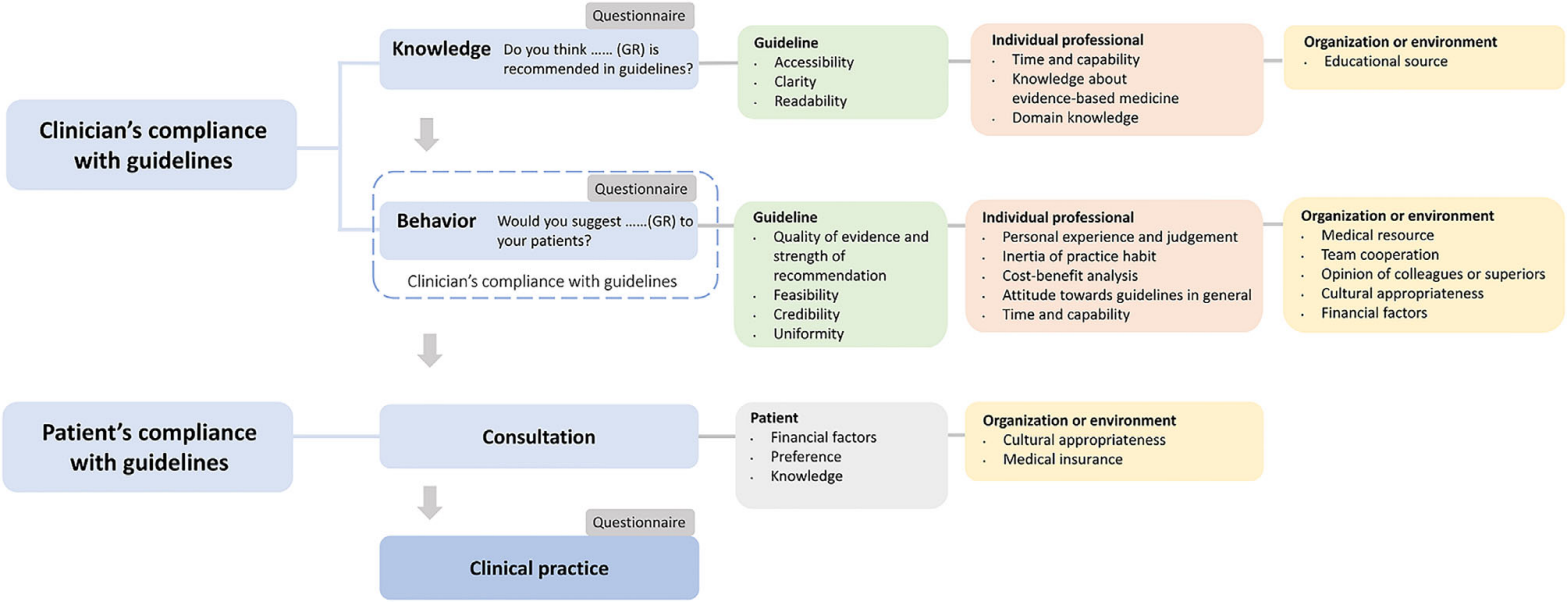
Sequence of clinician’s compliance and associated factors.

Besides, studies of similar themes or about determinants of practice were also reviewed to analyze the reason for non-compliance. After reviewing studies about compliance with the guidelines, determinants of practice, and others, we developed a flowchart to incorporate the sequence of compliance and corresponding influencing factors (**Figure 1**) (11, 14, 15).

## Discussion

Our findings demonstrated the discrepancy between guideline and practice, inadequate knowledge of guidelines, and deficient compliance with guidelines, all of which were also reported by previous studies (8–10). In the analysis of findings, we found that factors associated with guidelines, individual professionals, patients, organizations, and the environment all contributed to the transition from guideline to practice (11, 14).

### Guideline Self-Related Factors

Guidelines need to reflect current research (16), and one out of five recommendations could be out of date after 3 years (17). CMA amended and published their guideline last October, while its previous version was published in 2014. Given that it took time for the guideline to disseminate, the CMA guideline that was familiar to respondents when filling in questionnaires might be the 2014 version. From the aspect of timeliness, it could have less credibility compared with the EAU and NCCN guidelines, which were updated every year. After all, these results show an evident preference of respondents for native guideline. A survey conducted in China explored the barriers and enablers for the implementation of guidelines, which reported that 27.3% of their respondents encountered language barriers associated with English guidelines (18). Language might be an important reason for the gap of utilization between guidelines in Chinese and non-native language. Efforts should be devoted to increase readability of guideline for non-native speakers, and to provide translated executive summaries of guidelines could be a solution. Meanwhile, it requires guideline users to build trust in non-native guidelines and improve linguistic proficiency.

In addition, the feasibility of the guidelines is crucial for urologists to determine whether to recommend the guidelines to patients in actual clinical situations. As mentioned above, although some respondents knew that “an incomplete resection” was an indication for a second TURBT in the guidelines, they would not comply with it. Two respondents indicated that there was no objective criteria for TURBT to judge whether it was “a complete resection” or not. Hence, urologists were confused with this indication when applying guidelines. To enhance feasibility, guideline developers could give a definition to “complete resection” (for example, there was no residue with the naked eyes during operation or with cystoscopy), or detail the situations for the recommendation, such as “clear visual field in operation” and “the histopathology of the tumour base was negative”.

### Individual Professional-Related Factors

Knowledge of guideline is an essential prerequisite for compliance (11). For patients with low-risk NMIBC, it was stated in the guidelines that a single postoperative intravesical instillation was considered to be standard and complete treatment (19), while an additional chemotherapy course did not confer benefit but increase the risk of side effects (20). Our results indicated disturbing overuse of further intravesical instillations for low-risk patients, which reveals insufficient knowledge. Moreover, we found that respondents who referred to more guidelines were more likely to comply with guideline recommendations about the utilization of BCG for low-risk patients. Reminding urologists of the need to devote more time in acquiring guideline recommendations is essential. On the other hand, educational source about guidelines needs to be developed to benefit more urologists.

We got feedback from the respondents that the capability of communication with patients is of vital importance in clinical practice, especially when persuading patients to agree to have a second TURBT, or eschew further chemotherapy installations for low-risk NMIBC. Maintaining a harmonious relationship between patients and clinicians based on mutual trust and communication was essential (12).

Furthermore, the capability and training of urologists determined whether they could implement the recommendation in practice. In the study of Witjes et al., only 17.3% of intermediate-risk patients received BCG therapy in their group, which conformed to our results (10). Under the premise of the high toxicity and treatment cessation rate of BCG, one of the various barriers to its utilization was the lack of experience and confidence in the prevention and management of BCG-associated adverse events. As the management of BCG toxicity has been expounded in the EAU guideline (19) and practical recommendations for the prevention and management of adverse events had been acknowledged, these efforts might increase knowledge and finally contribute to clinical practice.

### Organization- or Environment-Related Factors

In our study, the lack of drug accessibility was the primary barrier to the utilization of BCG. Not only some respondents reported no local supply of BCG, but also the requirements for the transportation and storage of BCG are strict, which presents a challenge to the ability of a hospital to source the medicine. Actually, BCG shortage was a global problem, especially comparing with increasing demands (21). Desouky indicated that the ongoing clinical trials using BCG against COVID-19 could aggravate the shortage and influence our urology practice (22). Under the circumstances, both the AUA and NCCN provided strategies for urologists to help mitigate the conflicts, such as using chemotherapy as a substitute in intermediate-risk NMIBC patients when BCG was second-line therapy (20, 23).

### Patient-Related Factors

A survey conducted in Europe also reported wide overuse of additional adjuvant intravesical chemotherapy instillations for low-risk NMIBC patients (8). For patients without supporting medical knowledge, it was firmly believed that chemotherapy was an essential measure for the management of all tumors. If the tumor recurs or progresses, the urologist would be severely blamed by patients and their relatives for his “negative” attitude. Hence, medical education for patients was necessary to improve their knowledge of disease management, especially their knowledge of and confidence in the guidelines. At this point, some guidelines have included patients in their targeted users, or a specially designed patient version has been published.

Meanwhile, the financial resources of patients also influenced the implementation of recommendations. The price of BCG was much higher than chemotherapy, but it was included into the scope of reimbursement only in a few provinces of China. Our study showed that a second TURBT and radical cystectomy were also limited by the financial resource of patients. For instance, patients need to pay the cost of operation, postoperative urine collection bag, skin protection materials, and others. However, BCG therapy could achieve cost savings by decreasing the risks of local recurrence and its attendant treatments (24). A cost-effectiveness study found that the implementation of BCG intravesical therapy decreased costs by $3,900 per 5-year recurrence-free interval compared with the total costs attributable to recurrences (25).

### Strengths and Limitations

This is the first nationwide survey on the compliance of urologists with NMIBC guidelines in China, and it reveals the reasons for non-compliance in detail. The questionnaire development and distribution were carried out under the guidance of methodological experts. Through detailed analysis, it provided reliable information for guideline developers to improve the guideline implementation. However, this study still has some limitations that have to be considered. Recall bias might exist as the results were based on the subjective feedback of urologists, especially in the self-reported barriers to guideline implementation. Respondents might overstate the impact of patients and reduce their own responsibilities in non-compliance. Meanwhile, the results might be affected by the sample size of the questionnaire as well as the unknown response rate.

## Conclusions

This survey revealed that although the vast majority of urologists acknowledged the positive effects of guidelines, the knowledge of and compliance with some recommendations in NMIBC guidelines were still not high enough. It highlights the overuse of further intravesical instillations for low-risk NMIBC patients and the underuse of BCG for intermediate-risk and high-risk patients. Guideline factors (lack of readability and feasibility), individual professional factors (insufficient knowledge, lack of capability and training), organization or environment factors (lack of educational and medical resources), and patient factors (preference and financial sources) jointly resulted in the non-compliance.

## Data Availability Statement

The raw data supporting the conclusions of this article will be made available by the authors, without undue reservation.

## Ethics Statement

The studies involving human participants were reviewed and approved by the Ethics Committee of Wuhan University School of Medicine. The patients/participants provided their written informed consent to participate in this study.

## Author Contributions

Study concept and design: Y-HJ, D-QW, and X-TZ. Acquisition of data: Y-XS, Y-YW, S-YY, LY, and B-HL. Analysis and interpretation of data: QH, D-QW, and XH. Drafting of the manuscript: D-QW, QH, and Y-HJ. Critical revision of the manuscript for important intellectual content: XH, Y-HJ, and T-ZL. Obtaining funding: Y-HJ and X-TZ. Administrative, technical, or material support: LY and X-TZ. Supervision: T-ZL and X-TZ. All authors contributed to the article and approved the submitted version.

## Funding

This work was supported (in part) by the Planning Project of Innovation and Entrepreneurship Training of Undergraduate of Wuhan University (S2019303011), the National Natural Science Foundation of China (82174230), and the National Key Research and Development Program of China (Science and Technology Powers Economy 2020; 2016YFC0106300).

## Conflict of Interest

The authors declare that the research was conducted in the absence of any commercial or financial relationships that could be construed as a potential conflict of interest.

## Publisher’s Note

All claims expressed in this article are solely those of the authors and do not necessarily represent those of their affiliated organizations, or those of the publisher, the editors and the reviewers. Any product that may be evaluated in this article, or claim that may be made by its manufacturer, is not guaranteed or endorsed by the publisher.
