# Supplementary material for: Knowledge of and Compliance With Guidelines in the Management of Non-Muscle-Invasive Bladder Cancer: A Survey of Chinese Urologists
Source: Front Oncol. 2021 Oct 27;11:735704. doi: 10.3389/fonc.2021.735704 (PMC8580413; doi:10.3389/fonc.2021.735704)
Supplement: Supplementary file 1 [file DataSheet_1.docx]

Supplementary Material

**Appendix 1. Translated Version of Questionnaire Assessing Compliance with NMIBC Guideline**

**Information for informed consent**

**Project Title:** An Investigation of Non-muscle Invasive Bladder Cancer（NMIBC）Clinical Practice Guideline Adherence, Clinical Diagnosis and Treatment Situations

We would like to invite you to participate in this project. The information provided here can help you decide whether or not to participate in this research and give informed consent. Please read it carefully, and if you have any questions, please ask the researcher responsible for the research. Please note that your participation is voluntary. If you agree to participate in the study, please click "Agree" after the statement.

**Background and objective:** Clinical practice guidelines (CPG) aim to reduce the differences in the performance level of individual medical institutions and physicians, and to improve the quality of medical care. This study investigates the clinical situations of NMIBC clinical practice guideline adherence and clinical diagnosis and treatment in order to provide evidence for analyzing the status of guideline implementation and proposing guideline implementation strategies

**Privacy and confidentiality measures:** The data collection is completely anonymous and you do not need to sign this informed consent. In addition, in order to protect your privacy, the questionnaire will only be used for statistical analysis, not for publication or circulation.

**Researcher and Contact:** The Second Clinical College of Wuhan University Yinghui Jin (13612153066) Danqi Wang (18372689831)

**Disclaimer:** I have read the informed consent material carefully and have had the opportunity to ask questions. I understand that participation in this study is voluntary and I can choose not to participate in this study if I wish.

□Agree □Disagree

**Part 1. Demographic characteristics of urologists**

**Q1.1 Gender：**

□ Male □Female

**Q1.2 Age：**

________

**Q1.3 Level of hospital：**

□Tertiary hospital □Secondary hospital □Primary hospital

**Q1.4 Province：**

________

**Q1.5 Years in practice：**

________

**Q1.6 Education background：**

□Doctor □Master □Bachelor □College

**Q1.7 Professional title：**

□Senior title □Vice-senior title □Middle title □Primary title

**Q1.8 Have you** **ever participated in the development of guidelines?**

□Yes □No

**Q1.9 Do you** **agree that high-quality clinical practice guidelines help to standardize clinical diagnosis and treatment and improve medical quality：**

□Yes □No

**Q1.10 Please check** **the NMIBC guidelines developers which you usually consulted（multiple choice）：**

□National Institute for Health and Care Excellence (NICE)

□American Urological Association (AUA)

□European Association of Urology (EAU)

□National Comprehensive Cancer Network (NCCN)

□Japanese Urological Association (JUA)

□Canadian Urological Association (CUA)

□Chinese Medical Association (CMA)

□China International Exchange and Promotive Association for Medical and Health Care (CPAM)

□Others

**Part 2. Clinical utilization of chemotherapy and BCG**

**Q2.1.1 Did low-risk NMIBC patients agree to have intravesical chemotherapy instillations?**

□Induction instillations □Induction and maintenance instillations □No

**Q2.1.2** **If low-risk NMIBC patients agree to have intravesical chemotherapy instillations, how long does the course last?**

**Immediate instillations for** □1-3week(s) □4-8weeks □9 weeks and above

**Maintenance instillations for** □less than 6 months □6-12 months □more than 12 months

**Q2.2.1 The frequency of low-risk NMIBC patients to agree to have intravesical BCG instillations?**

□Never □Seldom □Sometimes □Often □Always

**Q2.2.2 If low-risk NMIBC patients agree to have intravesical BCG instillations, how long does the course last?**

**Induction instillations for** □1-5weeks □6-8weeks □more than 8 weeks

**Maintenance instillations for** □1week □2 weeks □3 weeks □4 weeks or more

**Time point** □the 3rd month □the 6th month □the 9th month □the 12th month □the 18th month □the 24th month □the 36th month

**Q2.2.3 If low-risk NMIBC patients agree to have intravesical BCG instillations, what is the recommended dose?**

□Reduced dose（1/3 standard dose） □Standard dose □Not clear

**Q2.3.1 Did intermediate-risk NMIBC patients agree to have intravesical chemotherapy instillations?**

□Induction instillations □Induction and maintenance instillations □No

**Q2.3.2 If** **intermediate-risk NMIBC patients agree to have intravesical chemotherapy instillations, how long does the course last?**

**Immediate instillations for** □1-3week(s) □4-8weeks □9 weeks and above

**Maintenance instillations for** □less than 6 months □6-12 months □more than 12 months

**Q2.4.1 The frequency of intermediate-risk NMIBC patients to agree to have intravesical BCG instillations?**

□Never □Seldom □Sometimes □Often □Always

**Q2.4.2 If intermediate-risk NMIBC patients agree to have intravesical BCG instillations, how long does the course last?**

**Induction instillations for** □1-5weeks □6-8weeks □more than 8 weeks

**Maintenance instillations for** □1week □2 weeks □3 weeks □4 weeks and above

**Time point** □the 3rd month □the 6th month □the 9th month □the 12th month □the 18th month □the 24th month □the 36th month

**Q2.4.3 If intermediate-risk NMIBC patients agree to have intravesical BCG instillations, what is the recommended dose?**

□Reduced dose（1/3 standard dose） □Standard dose □Not clear

**Q2.5.1 Did high-risk NMIBC patients agree to have intravesical chemotherapy instillations?**

□Induction instillations □Induction and maintenance instillations □No

**Q2.5.2 If high-risk NMIBC patients agree to have intravesical chemotherapy instillations, how long does the course last?**

**Immediate instillations for** □1-3week(s) □4-8weeks □9 weeks and above

**Maintenance instillations for** □less than 6 months □6-12 months □more than 12 months

**Q2.6.1 The frequency of high-risk NMIBC patients to agree to have intravesical BCG instillations?**

□Never □Seldom □Sometimes □Often □Always

**Q2.6.2 If high-risk NMIBC patients agree to have intravesical BCG instillations, how long does the course last?**

**Induction instillations for** □1-5weeks □6-8weeks □more than 8 weeks

**Maintenance instillations for** □1week □2 weeks □3 weeks □4 weeks and above

**Time point** □the 3rd month □the 6th month □the 9th month □the 12th month □the 18th month □the 24th month □the 36th month

**Q2.6.3 If high-risk NMIBC patients agree to have intravesical BCG instillations, what is the dose?**

□Reduced dose（1/3 standard dose） □Standard dose □Not clear

**Part 3. Knowledge of guidelines**

**Q3.1 Was “the presence of detrusor muscle in the specimen is necessary in initial TURBT” recommended by guidelines?**

□Yes □No □Not clear □Beyond my scope of clinical practice

**Q3.2 Was a single postoperative instillation of intravesical chemotherapy after TURBT recommended by guidelines?**

□Yes □No □Not clear □Beyond my scope of clinical practice

**Q3.3 For low-risk NMIBC patients, was intravesical chemotherapy instillations recommended by guidelines?**

□Induction instillations □Induction and maintenance instillations □No □Not clear

□Beyond my scope of clinical practice

**Q3.4 For low-risk NMIBC patients, was intravesical BCG instillations recommended by guidelines?**

□Induction instillations □Induction and maintenance instillations □No □Not clear

□Beyond my scope of clinical practice

**Q3.5 For intermediate-risk NMIBC patients, was intravesical chemotherapy instillations recommended by guidelines?**

□Induction instillations □Induction and maintenance instillations □No □Not clear

□Beyond my scope of clinical practice

**Q3.6 For intermediate-risk NMIBC patients, was intravesical BCG instillations recommended by guidelines?**

□Induction instillations □Induction and maintenance instillations □No □Not clear

□Beyond my scope of clinical practice

**Q3.7 For high-risk NMIBC patients, was intravesical BCG instillations recommended by guidelines?**

□Induction instillations □Induction and maintenance instillations □No □Not clear

□Beyond my scope of clinical practice

**Q3.8** **Second TURBT** **was recommended by guidelines for patients in the following situations…?**

□After incomplete initial TURBT

□There is no muscular layer in the first resected specimen (except for TaLG / G1 tumor and carcinoma in situ)

□In T1 tumours

□In G3/high-grade tumours (except for CIS)

□Pathology analysis results of initial TURBT failed to determine stage or risk grading

□Others ________________

□Not clear

□Beyond my scope of clinical practice

**Q3.9 Radical cystectomy was recommended by guidelines for patients in the following situations****…?**

□High-grade T1 with histological variation (micropapillary, sarcoma, small cell type)

□High-grade T1 with lymphatic vessel infiltration, multiple and/or large high-grade T1, high-grade T1 with bladder/prostate CIS

□Pathology analysis results of a second TURBT is still high-grade T1

□High-grade NMIBC with early recurrence within 3 months

□NMIBC involving the bladder diverticulum

□High-risk NMIBC patients with BCG failure*

□Tumor Involving the distal urethra or / and the prostate urethra, tumor located in the endoscope field of anatomical blind area

□Not clear

□Beyond my scope of clinical practice

**Part 4. Compliance with guidelines**

**Q4.1 Would you suggest performing intravesical chemotherapy instillations for low-risk NMIBC patients?**

□Induction instillations □Induction and maintenance instillations □No □Not clear

□Beyond my scope of clinical practice

**Q4.2 Would you suggest performing intravesical BCG instillations for low-risk NMIBC patients?**

□Induction instillations □Induction and maintenance instillations □No □Not clear

□Beyond my scope of clinical practice

**Q4.3 Would you suggest performing intravesical chemotherapy instillations for intermediate-risk NMIBC patients?**

□Induction instillations □Induction and maintenance instillations □No □Not clear

□Beyond my scope of clinical practice

**Q4.4 Would you suggest performing intravesical BCG instillations for intermediate-risk NMIBC patients?**

□Induction instillations □Induction and maintenance instillations □No □Not clear

□Beyond my scope of clinical practice

**Q4.5 Would you suggest performing intravesical chemotherapy instillations for high-risk NMIBC patients?**

□Induction instillations □Induction and maintenance instillations □No □Not clear

□Beyond my scope of clinical practice

**Q4.6 Would you suggest performing intravesical BCG instillations for high-risk NMIBC patients?**

□Induction instillations □Induction and maintenance instillations □No □Not clear

□Beyond my scope of clinical practice

**Q4.7 Would you suggest a second TURBT to patients in the following situations…?**

□After incomplete initial TURB

□There is no muscular layer in the first resected specimen (except for TaLG / G1 tumor and carcinoma in situ)

□In T1 tumours.

□In G3/high-grade tumours(except for CIS)

□Pathology analysis results of initial TURBT failed to determine stage or risk grading

□Others ________________

□Not clear

□Beyond my scope of clinical practice

**Q4.8 Would you suggest radical cystectomy to patients in the following situations …?**

□High-grade T1 with histological variation (micropapillary, sarcoma, small cell type)

□High-grade T1 with lymphatic vessel infiltration, multiple and/or large high-grade T1, high-grade T1 with bladder/prostate CIS

□Pathology analysis results of a second TURBT is still high-grade T1

□High-grade NMIBC with early recurrence within 3 months

□NMIBC involving the bladder diverticulum

□High-risk NMIBC patients with BCG failure*

□Tumor involving the distal urethra or / and the prostate urethra, tumor located in the endoscope field of anatomical blind area

□Not clear

□Beyond my scope of clinical practice

* Supplementary information: BCG treatment failure can be considered when one of the following conditions occurs in a BCG-treated patient:

1. Muscle-invasive tumour appears during or after BCG therapy.

2. High-grade tumour is present during BCG therapy.

3. High-grade recurrence after BCG.

4. Severe side effects that prevent further BCG instillation before completing treatment.

Patients with low-grade recurrence during or after BCG treatment are not considered to be a BCG failure.

**Part 5. Barriers to guideline implementation**

**Q5.1 Barriers to the implementation of second TURBT**

□Urologist didn’t suggest it because of because of risk or complications

□Patients rejected the operation because of risk or complications

□Patients rejected the operation for economic reasons

□Others_______________

**Q5.2 Barriers to the implementation of BCG**

**Drug was not accessible:** □Yes □No

**Urologist didn’t suggest it** **because of risk or side effects:** □Never □Seldom □Sometimes □Often □Always

**Patients rejected the operation for economic reasons:** □Never □Seldom □Sometimes □Often □Always

**Patients rejected it because of risk or side effects:** □Never □Seldom □Sometimes □Often □Always

**Q5.3 Barriers to the implementation of radical cystectomy**

**Patients rejected the operation for economic reasons:** □Never □Seldom □Sometimes □Often □Always

**Patients rejected it because of decrease in life quality:** □Never □Seldom □Sometimes □Often □Always

**Patients rejected it for personal reasons:** □Never □Seldom □Sometimes □Often □Always

**Patients rejected it because of risk or complications:** □Never □Seldom □Sometimes □Often □Always

**Appendix 2. Strategies of further intravesical chemotherapy and BCG instillations**

|  | Induction Course | | | | Maintenance Course | | | | |
| --- | --- | --- | --- | --- | --- | --- | --- | --- | --- |
| Chemotherapy | 1-3 weeks | 4-8 weeks | 9 weeks and above | Total | less than 6 months | | 6-12 months | more than 1 year | Total |
|  | 141 | 383 | 264 | 788 | 66 | | 434 | 134 | 634 |
|  | 82 | 390 | 308 | 780 | 54 | | 461 | 214 | 729 |
|  | 70 | 340 | 322 | 732 | 42 | | 330 | 321 | 693 |
| BCG | 1-5w | 6-8w | more than 8 weeks | Total | 1 w | 2w | 3w | 4w and above | Total |
|  | 164 | 223 | 134 | 521 | 78 | 32 | 17 | 207 | 334 |
|  | 130 | 284 | 176 | 590 | 76 | 42 | 39 | 318 | 475 |
|  | 99 | 269 | 245 | 613 | 81 | 52 | 42 | 389 | 564 |

**Appendix 3. Barriers to the implementation of guideline recommendations**

| Potential Barriers | Second TURBT | BCG | RC |
| --- | --- | --- | --- |
| Urologist didn’t suggest it because of risk or side effects | 37.55% | 26.00% | - |
| Patients rejected it because of risk or side effects | 70.63% | 62.22% | 38.14% |
| Patients rejected the operation for economic reasons | 50.43% | 40.67% | 29.21% |
| Drug was not accessible | - | 74.89% | - |
| Patients rejected it because of decrease in life quality | - | - | 57.73% |
| Patients rejected for personal reasons | - | - | 47.42% |
